# Coordinately Co-opted Multiple Transposable Elements Constitute an Enhancer for *wnt5a* Expression in the Mammalian Secondary Palate

**DOI:** 10.1371/journal.pgen.1006380

**Published:** 2016-10-14

**Authors:** Hidenori Nishihara, Naoki Kobayashi, Chiharu Kimura-Yoshida, Kuo Yan, Olga Bormuth, Qiong Ding, Akiko Nakanishi, Takeshi Sasaki, Mika Hirakawa, Kenta Sumiyama, Yasuhide Furuta, Victor Tarabykin, Isao Matsuo, Norihiro Okada

**Affiliations:** 1 Department of Life Science and Technology, Tokyo Institute of Technology, Midori-ku, Yokohama, Kanagawa, Japan; 2 Osaka Medical Center and Research Institute for Maternal and Child Health, Osaka Prefectural Hospital Organization, Izumi, Osaka, Japan; 3 Institute of Cell Biology and Neurobiology, Charité–Universitätsmedizin Berlin, Berlin, Germany; 4 Bioinformatics Center, Institute for Chemical Research, Kyoto University, Gokasho, Uji, Kyoto, Japan; 5 National Institute of Genetics, Mishima, Shizuoka, Japan; 6 Animal Resource Development Unit, RIKEN Center for Life Science Technologies, Chuou-ku, Kobe, Japan; 7 Genetic Engineering Team, RIKEN Center for Life Science Technologies, Chuou-ku, Kobe, Japan; 8 Department of Life Sciences, National Cheng Kung University, Tainan, Taiwan; 9 Foundation for Advancement of International Science, Tsukuba, Japan; University of Utah School of Medicine, UNITED STATES

## Abstract

Acquisition of *cis*-regulatory elements is a major driving force of evolution, and there are several examples of developmental enhancers derived from transposable elements (TEs). However, it remains unclear whether one enhancer element could have been produced via cooperation among multiple, yet distinct, TEs during evolution. Here we show that an evolutionarily conserved genomic region named AS3_9 comprises three TEs (AmnSINE1, X6b_DNA and MER117), inserted side-by-side, and functions as a distal enhancer for *wnt5a* expression during morphogenesis of the mammalian secondary palate. Functional analysis of each TE revealed step-by-step retroposition/transposition and co-option together with acquisition of a binding site for Msx1 for its full enhancer function during mammalian evolution. The present study provides a new perspective suggesting that a huge variety of TEs, in combination, could have accelerated the diversity of *cis*-regulatory elements involved in morphological evolution.

## Author Summary

Acquisition of *cis*-regulatory elements is a major driving force of evolution, but its whole evolutionary significance is poorly understood. Here, we found an unprecedented case in that three TEs cooperatively and synergistically act as a distal enhancer for *wnt5a* expression in mammalian frontonasal region, being involved in the evolution of the secondary palate formation. We elucidated the stepwise evolutionary history of getting the enhancer activity via (retro-)transposition of TEs during mammalian evolution. Taking various types of TEs constituting nearly half of mammalian genomes into consideration, this study provides a new perspective for the potentiality that almost infinite combinations of proximally-located different TEs could have generated a huge diversity of developmental enhancers and had a great impact on mammalian morphological evolution.

## Introduction

Morphogenesis is generally controlled by spatiotemporal expression of a number of specific gene sets [1]. The acquisition of novel phenotypic traits during mammalian evolution has been posited to result from changes in gene expression patterns, which are mediated by gain of new *cis*-regulatory elements such as enhancers [2]. Mammalian genomes contain hundreds of thousands of conserved non-coding elements (CNEs), which, in humans, occupy 3–8% of the genome [3]. Because CNEs are expected to include a number of transcriptional enhancers [4], they are recognized as highly important clues to understanding the key gene regulatory mechanisms involved in mammalian evolution [5–8].

Mammals have acquired a variety of morphological features during evolution. One of the striking evolutionary events is the development of the bony secondary palate [9]. Complete closure of the mammalian secondary palate during development (palatogenesis) separates the oral cavity from the nasal cavity, which allows breathing while eating and efficient suckling. This closure begins with the formation of bilateral palatal shelves (PS) in the embryonic maxillary prominences, and then the PS grow horizontally to fuse with each other at the midline [10] (S1 Fig). Dozens of genes such as the *msx1* and *wnt* family are known to be involved in palatogenesis [11, 12]. Agenesis of the secondary palate, known as cleft lip/palate, is one of the most common congenital defects in humans, occurring once in every 700 newborns [11]. In this regard, *wnt5a* is one of the possible responsible genes identified by genetic association studies of human cleft lip/palate [13]. Correspondingly, mice lacking *wnt5a* or its non-canonical receptor *Ror2* are born with cleft palate [14, 15]. Therefore, revealing the molecular regulatory mechanisms of such genes, which remain largely unknown, is essential to our understanding of the molecular basis of mammalian-specific morphological evolution as well as that of the cleft lip/palate defect in humans.

Transposable elements (TEs), i.e., retroposons and DNA transposons, occupy nearly half of mammalian genomes. Retroposons such as SINEs propagate their copies via reverse-transcription of their RNA intermediates, with reintegration of the copied DNA, whereas DNA transposons simply directly relocate within the genome [16–18]. Although TEs are, in general, regarded as genomic parasites or sometimes as harmful dynamic mutagens, we for the first time proposed, together with the Bejerano’s group, that some TEs are involved in macro-evolution by showing that they overlap with CNEs [7, 19]. This fact implies that TEs under purifying selection acquired functions during evolution [20, 21], which is called exaptation [22] or co-option, and that many types of TEs such as SINEs might have contributed to various morphological innovations during mammalian evolution [8]. Indeed, we previously demonstrated that hundreds of AmnSINE1 sequences are evolutionarily conserved among mammals [7, 23, 24]. One AmnSINE1 is an enhancer of *fgf8* in the diencephalon, and another acts as an enhancer of *satb2* expression in the deep layer of the neocortex, especially in callosal projection neurons [23, 25, 26]. Further, the LF-SINE locus, which is shared among tetrapods, serves as a distal enhancer of the neurodevelopmental gene *Isl1* [19], and *Pomc* has two neuronal enhancers derived from CORE-SINE and MaLR [27, 28]. Thus, it has been clearly established that TEs are one of the main sources of *cis*-regulatory elements [29].

Mammalian genomes harbor a variety of TEs as exemplified by the human genome, which has >1,100 types (subfamilies) that occupy >45% of the genome. These facts prompted us to consider whether multiple TEs of different origin and sequence, being located proximal to one another, could be co-opted/exapted as a single enhancer element. If so, a huge diversity of developmental enhancers could have been generated by combining different TE types during evolution. This possibility has never been examined, however, and in all the known cases of co-option/exaptation, a developmental enhancer was found to consist of only a single TE [19, 23, 27, 28], although one interesting case in which the promoter region of decidual prolactin was reported to be derived from two TEs [30].

Here, we report that a CNE containing three TEs, including AmnSINE1, acts as a distal enhancer of *wnt5a* during palatogenesis. This is an unprecedented example, to our knowledge, in which three different TEs inserted side-by-side play a cooperative role in the distal enhancer function within a CNE. TEs located proximal to one another may have potential as genetic sources of diversity of regulatory elements and that such cooperative enhancers might have contributed to mammalian morphological evolution through controlling spatiotemporally diverse expression of various genes.

## Results

### AS3_9 locus as a distal enhancer of *wnt5a* in the embryonic frontonasal region including PS

**Fig 1:**
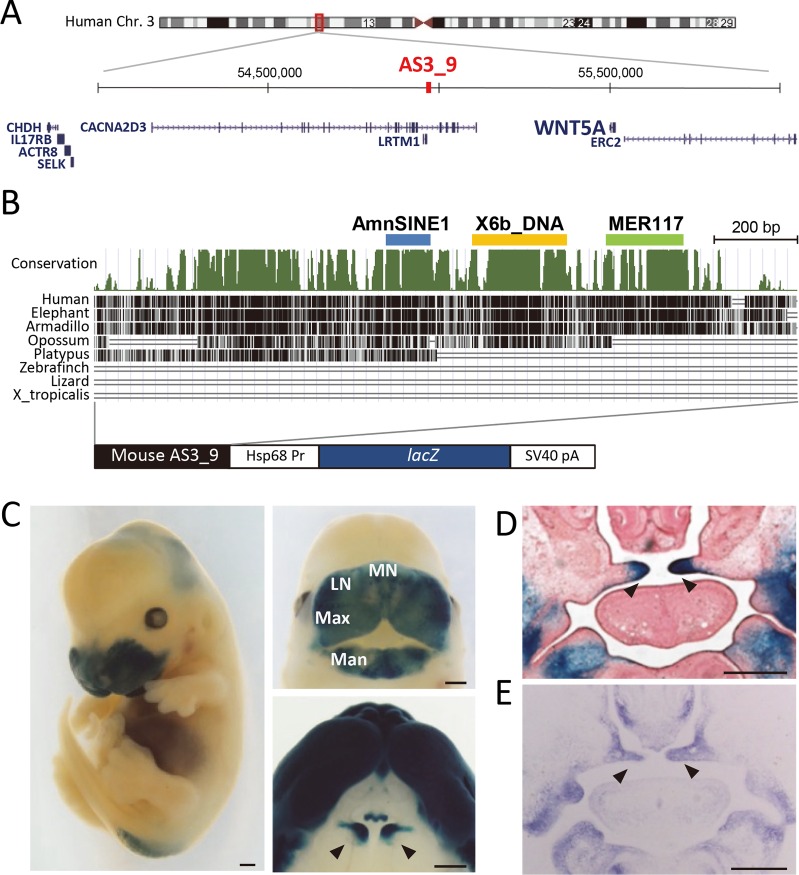
AS3_9 locus and its enhancer activity in the frontonasal region. (A) Location of human AS3_9 and surrounding genes. (B) The phastCons conservation track of AS3_9 in mouse (UCSC Genome Browser; mm10); the blue, yellow, and green bars denote the AmnSINE1, X6b_DNA, and MER117 regions, respectively. The construct used to produce AS3_9-*lacZ* mice is illustrated below. (C) Whole-mount (left), frontal view of the head (top right), and ventral view of the upper jaw (bottom right) showing *lacZ* expression in AS3_9-*lacZ* E13.5 embryos. (D) Coronal section of an AS3_9-*lacZ* E14.5 embryo. (E) ISH for *wnt5a* in E14.5 wild type. Arrowheads indicate the palatal shelves (C–E). MN, medial nasal process; LN, lateral nasal process; Max, maxillary process; Man, mandibular process. Scale bar (C–E): 0.5 mm.

The 1.2-kb AS3_9 locus is located at chr3:54916774–54917973 of the human genome (GRCh38/hg38) (Fig 1A and 1B). This locus is one of the hundreds of AmnSINE1-derived CNEs in mammals identified by our group [24]. In this locus, the AmnSINE1-related region is 71.4% identical to nucleotide positions 391–501 of its original consensus sequence (Fig 1B, S2 Fig) [7]. To test whether the evolutionarily conserved AS3_9 locus possesses an enhancer function—as is the case for other AmnSINE1-derived CNEs [23, 25, 26]—we performed a transgenic mouse enhancer analysis using a construct containing AS3_9 and a *lacZ* reporter gene (Fig 1B). The transgenic mice (AS3_9-*lacZ*) consistently displayed strong *lacZ* expression in the frontonasal region at embryonic day 13.5 (E13.5) (Fig 1C, S3A Fig). Especially, *lacZ* was expressed in the frontonasal prominence including the medial and lateral nasal processes, the maxillary processes that give rise to the upper lip and PS, and mandibular processes that form the lower lip. The *lacZ* expression patterns in the frontonasal prominence during embryogenesis were consistent among the three AS3_9-*lacZ* mouse lines we established in this study (S3B Fig).

We expected that this TE-derived CNE serves as a distal enhancer of a gene responsible for the development of the frontonasal region in mammals. The ~2-Mb region surrounding AS3_9 contains eight genes (Fig 1A). We carried out *in situ* hybridization (ISH) for each of the eight candidate genes by using the respective mRNA as a probe; this revealed that only *wnt5a* is expressed in the frontonasal region (S4 Fig). This is consistent with a previous report that *wnt5a* is expressed in the frontonasal prominence and anterior side of PS [14] and is responsible for secondary palate development [12, 15]. We also found that *lacZ* expression in the AS3_9-*lacZ* embryos coincided exactly with *wnt5a* expression in the frontonasal prominence at E10.5 (S5A Fig) as well as in the anterior side of PS at E13.5–14.5 (Fig 1C–1E; S5B Fig). These results suggested that AS3_9 is a distal enhancer for *wnt5a* expression in the frontonasal region including PS.

### Abnormal expression of *wnt5a* in AS3_9-knockout (AS3_9-ko) mice

**Fig 2:**
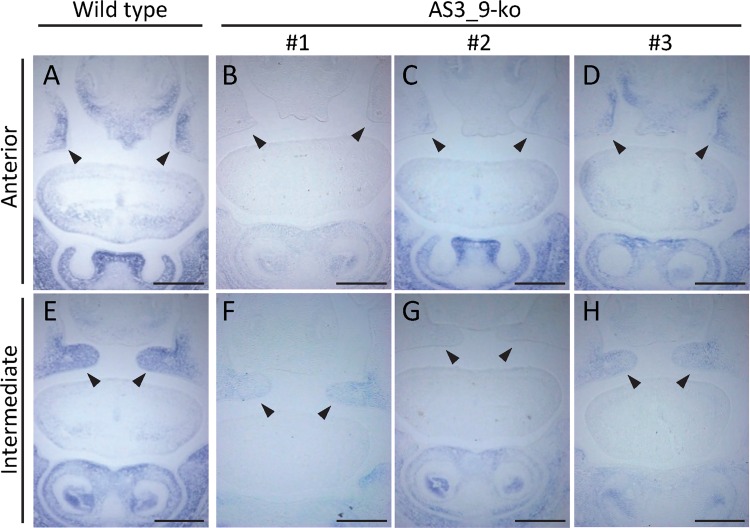
*Wnt5a* expression in E14.5 coronal sections from the anterior and intermediate regions of the oral cavity in one wild-type and three AS3_9-ko embryos (#1–3). (A) Anterior region of a wild-type embryo. (B-D) Anterior region of three AS3_9-ko embryos. (E) Intermediate region of a wild-type embryo. (F-H) Intermediate region of three AS3_9-ko embryos. Arrowheads denote palatal shelves. Scale bar: 0.5 mm.

We next assessed whether AS3_9 serves as an enhancer of *wnt5a* expression during secondary palate formation. Using AS3_9-ko mouse embryos in which the TE-derived 800-bp region of AS3_9 was targeted (see S6 Fig), we carried out both ISH (*wnt5a* mRNA probe) and a histological analysis. AS3_9 homozygous mutant mice established from two lines were viable and fertile. Expression of *wnt5a* in the frontonasal region of E14.5 homozygous AS3_9-ko embryos was weak and/or irregular compared with wild-type littermates (Fig 2). For example, one of the AS3_9-ko mice (#1 in Fig 2) showed no *wnt5a* expression on the anterior side of PS (Fig 2B) and very weak expression in the PS and mandibular processes in the intermediate region (Fig 2F). Another mouse (#2) displayed moderate *wnt5a* expression in mandibular processes but little in the PS (Fig 2C and 2G). Therefore, none of the AS3_9-ko embryos showed strong *wnt5a* expression, i.e., equivalent to that of wild type. This result demonstrated that AS3_9 is indeed an enhancer of *wnt5a* expression. To investigate whether reduced *wnt5a* expression could affect PS development, we performed a histological analysis of E14.5 wild-type and AS3_9-ko littermates; notably, the knockout embryos did not exhibit any distinguishable agenesis or delayed palatogenesis (S7A Fig). Moreover, at E15.5, the PS of AS3_9-ko embryos were completely closed as was observed in the wild-type embryos (S7B Fig). Therefore, even when *wnt5a* expression was unstable or weak in the AS3_9 mutants, palatogenesis progressed essentially normally, presumably owing to a compensation mechanism involving other cis-elements (see Discussion).

### AS3_9 contains three TEs that play different roles in the enhancer function

**Fig 3:**
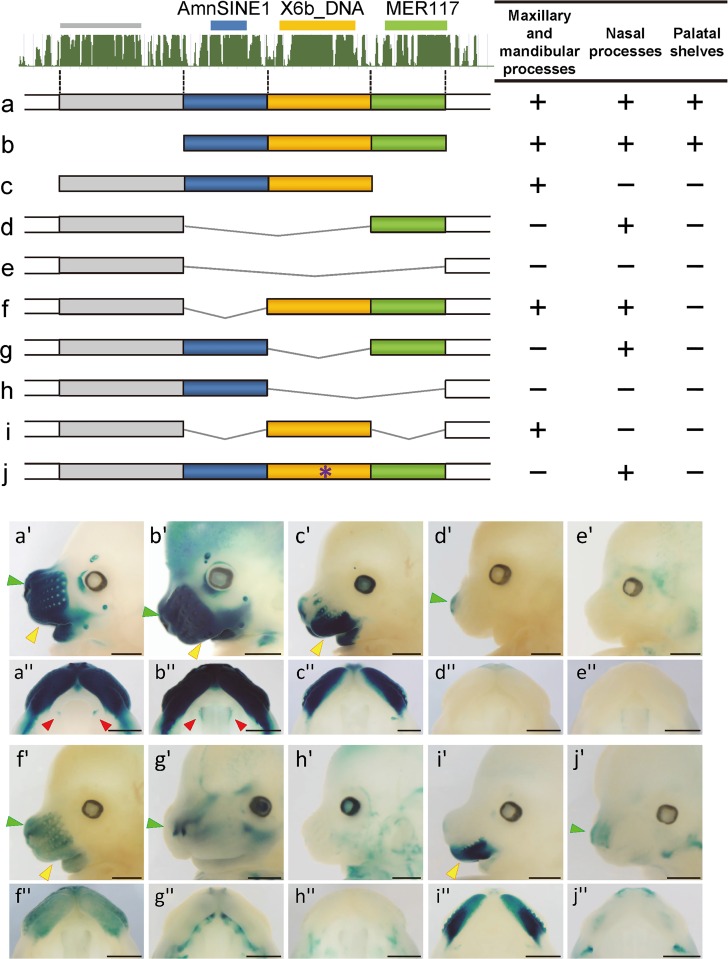
Functional dissection of AS3_9 enhancer. Top left: Conservation track from the UCSC Genome Browser (mm10) and TE regions in mouse. Shown are nine constructs (a–j) and their enhancer activity in the maxillary and mandibular processes, nasal processes, and palatal shelves (right). Lateral view (a'–j') and ventral view (a''–j'') of the upper jaw of E13.5 transgenic embryos harboring each deletion construct (a–i) and the construct with a mutated Msx1-binding site (j). *LacZ* expression was clearly observed in the maxillary and mandibular processes (yellow arrowheads), nasal processes (green arrowheads), and palatal shelves (red arrowheads). Scale bar: 1 mm.

The conservation pattern of AS3_9 implies that this CNE can be divided into four sub-elements (conservation graph in Fig 1B), prompting us to investigate the origins of the conserved sub-elements. Interestingly, we found that, in addition to the AmnSINE1 region, two other conserved sub-elements were derived from other TEs, namely X6b_DNA and MER117, which are 74.4% and 72.8% identical to their full-length consensus sequences, respectively (S8A and S8B Fig). X6b_DNA is a non-autonomous DNA transposon distributed in Theria (placental mammals and marsupials), whereas MER117 is a hAT-type non-autonomous DNA transposon distributed only in placental mammals. Consistent with these distributions, we found that the orthologs of the X6b_DNA and MER117 elements in AS3_9 are only found in therian and placental mammals, respectively (Fig 1B; S8 Fig). The other conserved region is not derived from a known TE or repetitive sequences (gray bar in Fig 3), as we confirmed that a RepeatMasker analysis and a blast search against the human genome returned no significant hit.

Because each of the three TEs in AS3_9 was conserved as an independent sub-element of the locus (S2 and S8 Figs), we expected that they make distinct contributions to overall enhancer function. To clarify each role, deletion constructs lacking various combinations of the TE regions were used for enhancer assays with transgenic mice (Fig 3A–3I, S9A–S9I Fig). At E13.5, enhancer activities were evaluated based on *lacZ* expression in the ventral region including the maxillary and mandibular processes, in the rostral region including the medial and lateral nasal processes, and in PS. Embryos harboring a construct with only the three TE regions (Fig 3B and 3B') showed strong *lacZ* expression equivalent to that of AS3_9-*lacZ* mice (Fig 1C, Fig 3A and 3A'). Conversely, the construct lacking the three TE-derived regions lacked enhancer activity in the frontonasal region (Fig 3E and 3E'). These results indicated that the three TE regions were sufficient to recapitulate the full AS3_9 enhancer activity and that other regions, such as the non-TE conserved region (gray bar in Fig 3), probably do not contribute to enhancer function. Constructs lacking the MER117 region showed no or very weak enhancer activity in the medial and lateral nasal processes (Fig 3C and 3C'), whereas only the constructs carrying MER117 yielded a *lacZ* signal in the apex of the nose (compare Fig 3D' with 3E', 3F' with 3I' and 3G' with 3H'). The MER117 region is, therefore, responsible for enhancer function in nasal processes, especially in the nose apex. The presence of X6b_DNA in the constructs always yielded *lacZ* expression in the ventral region, namely, the maxillary and mandibular processes (Fig 3A', 3B', 3C', 3F' and 3I'). Consistently, only the lack of X6b_DNA resulted in no *lacZ* expression in this region (compare Fig 3A' with 3G', 3D' with 3F' and 3E' with 3I'). Accordingly, the X6b_DNA region is responsible for the enhancer activity in the ventral region. The AmnSINE1 region alone did not yield *lacZ* expression (Fig 3H and 3H'); however, this region increased the range and intensity of the enhancer activity of X6b_DNA and MER117. For example, X6b_DNA alone supported enhancer activity mainly in the maxillary process and weak activity in the mandibular process (Fig 3I and 3I'); addition of AmnSINE1 yielded strong *lacZ* expression in the mandibular process as well as limited parts of the nasal prominence (Fig 3C and 3C'). Likewise, when AmnSINE1 was present, the enhancer signal of MER117 at the nose apex (Fig 3D') extended somewhat further toward the upper region of the medial nasal processes and part of the lateral nasal processes (Fig 3G and 3G'). Notably, enhancer activity in PS was observed only when all three TE regions were included in the same construct (Fig 3A'' and 3B''). These results suggested that each TE plays a distinct role in *wnt5a* enhancer function.

### Msx1 binds AS3_9

To elucidate the molecular mechanism of the AS3_9 enhancer, we utilized the yeast one-hybrid system to search for transcription factors that bind the AS3_9 sequence. Twelve candidate genes were identified (S1 Table, S10A–S10C Fig), of which three (Msx1, Msx2, Gtf2ird1) are known to be involved in mammalian craniofacial development [31, 32]. The most noteworthy finding was *msx1* because it has been demonstrated as one of the genes responsible for cleft palate in humans [33] and mice [31, 34, 35]. We found an Msx1-binding motif (TAATTG) [36] within the X6b_DNA-derived sequence of AS3_9. Mutation of this site abrogated Msx1 binding (S10D–S10F Fig). Furthermore, we conducted enhancer analysis using the AS3_9 sequence in which an identical mutation was introduced in the Msx1-binding site. Intriguingly, the transgenic embryos showed limited enhancer activity in the medial nasal process and maxillary process as well as loss of activity in PS (Fig 3J', S9J Fig), similar to the X6b_DNA-deleted constructs (Fig 3D' and 3G'). These results indicated that Msx1-binding is essential for the full enhancer function of AS3_9 and suggested that the *msx1* and *wnt5a* signaling pathways may interact closely during the secondary palate development.

## Discussion

**Fig 4:**
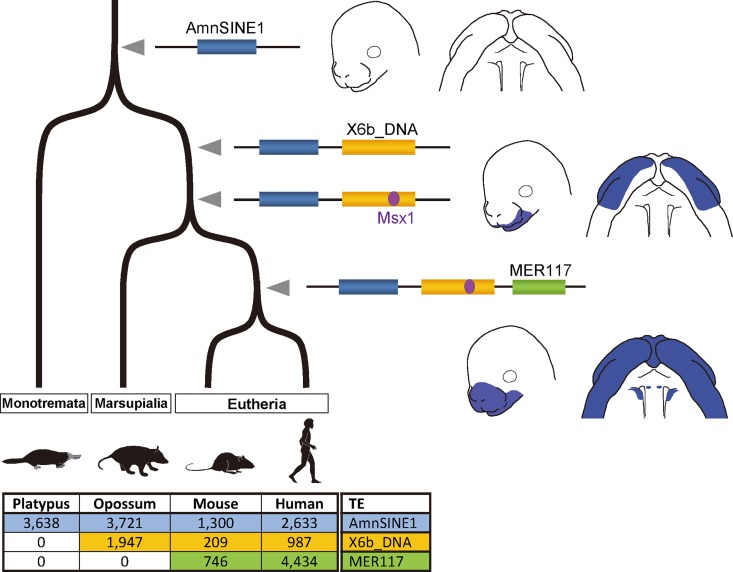
Evolution of the AS3_9 enhancer. AmnSINE1 (blue), X6b_DNA (yellow) and MER117 (green) were (retro)transposed side by side, and the enhancer had been gradually established. Copy numbers of the TEs in the representative genomes are shown below, indicating their clade-specific distributions.

Our analysis of the orthologs within the AS3_9 locus revealed that the AmnSINE1, X6b_DNA, and MER117 elements are present only among Mammalia, Theria, and Eutheria, respectively, suggesting that they were integrated in this order during evolution (Fig 1A, S2 and S8 Figs). Fig 4 shows the evolutionary scenario for the establishment of the AS3_9 enhancer. Although the AmnSINE1-derived region alone lacks enhancer function, this region is evolutionarily conserved even between humans and platypus (S2 Fig). Therefore, the AmnSINE1-derived region might have had another/unknown function in a common ancestor of mammals before 186 million years ago (Mya) [37], and it might have acquired a new additional role as the AS3_9 enhancer during evolution. After divergence of monotremes, integration of X6b_DNA and subsequent acquisition of the Msx1-binding site resulted in co-option/exaptation 170–186 Mya [37]. The AmnSINE1 and X6b_DNA region might serve as the *wnt5a* enhancer in the developing maxillary and mandibular processes. After divergence of marsupials (170 Mya) [37], MER117 was integrated, and finally AS3_9 was established as the current complete enhancer with extended activity to the medial and lateral nasal processes as well as PS. This is the first demonstration, to our knowledge, of stepwise evolution via co-option/exaptation of a developmental enhancer.

The full activity of the AS3_9 enhancer in the whole frontonasal region and PS was not observed with any one of AmnSINE1, X6b_DNA, and MER117 alone and was only attained with the combination of all three sub-elements (Fig 3A'' and 3B''). Therefore, the three TEs act cooperatively and synergistically as one complex distal enhancer element during palatogenesis. The division of roles among the TEs (Fig 3) implies that they undergo different epigenetic modifications in the different tissues. To address this possibility, we investigated the ChIP-seq data of the ENCODE project available in the UCSC database (S11A Fig) and the Roadmap Epigenomics project data (S11B Fig; http://www.roadmapepigenomics.org/). The UCSC genome browser shows that the chromatin states of AmnSINE1 and X6b_DNA regions of AS3_9 are open in various fibroblast cell lines (black bar in S11A Fig). This strong signal for open chromatin in the X6b_DNA region is also observed in the Roadmap Epigenomics data (see DNase column in S11B Fig). For histone modifications, the AmnSINE1 and MER117 regions show weak inactive/heterochromatin states (e.g., H3K9me3/H3K27me3/H3K36me3) in many other cells (S11B Fig). The ChIP-seq data for transcription factors show the binding of CTCF to the AmnSINE1 + X6b_DNA region and the bindings of NR2C2 and SRF to the MER117 (S11A Fig). Although involvement of NR2C2 or SRF in palatogenesis has not been reported, it is possible that these proteins are involved in the secondary palate formation in mammals. Unfortunately, these epigenetic states has not been tested in the frontonasal region or PS during the corresponding developmental stages of mice. Future examination for these epigenetic signals of AS3_9 may lead us to further understanding of the molecular mechanism for the formation of the secondary palate in mammals.

It is generally considered that robust gene expression is ensured by the presence of a backup *cis*-regulatory system such as primary and secondary enhancers [38, 39]. Actually, many studies have demonstrated that deletion of one enhancer can perhaps be compensated by another enhancer with little effect on phenotype [40–42]. Therefore, complete palatogenesis in the AS3_9-ko mice was probably due to the remaining of weak *wnt5a* expression (Fig 2, S7 Fig). It is likely that AS3_9 serves as one of multiple *cis*-regulatory elements responsible for *wnt5a* expression during secondary palate development. This hypothesis can be rationalized from paleontological evidence that suggests that acquisition of the bony secondary palate dates back 200 Mya [43]. Therefore, before the divergence of monotremes (184 Mya), other enhancer(s), the presence of which was suggested above, might have been responsible for the formation of the bony secondary palate of early mammals.

Because *wnt5a*-deficient mice reduced expression of *msx1*, *bmp4*, and *shh* in the anterior palate, *wnt5a* is considered to act upstream of these genes [12, 15]. Little is known, however, about the molecular mechanisms by which *wnt5a* expression is regulated. *Msx1* is also associated with human non-syndromic cleft palate [33], and *msx1*-deficient mice have cleft secondary palate [34] as well as reduced expression of *bmp4* and *shh* [35]. In the anterior PS, *msx1* up-regulates *bmp4* and vice versa, and *bmp4* controls the downstream *shh* signaling that triggers PS growth [12, 35]. As we showed in the present study, it is remarkable that the Msx1-binding site in AS3_9 is necessary for its enhancer function (Fig 3J and 3J', S9J Fig), suggesting that *wnt5a* expression in the anterior palate is controlled by *msx1*. Therefore, taking the previous study suggesting that *msx1* is one of the downstream genes of *wnt5a* into consideration [15], *wnt5a* and *msx1* may have a synergistic effect on palatogenesis, as is the case with *msx1* and *bmp4* [35]. Therefore, palatogenesis is presumably controlled not by simple hierarchical signaling but rather by various interdependent *cis*-regulatory elements.

The present study showed that in the distal enhancer of *wnt5a* three TEs take their part cooperatively in palatogenesis (Fig 3). Notably, each of the X6b_DNA and MER117 regions of AS3_9 possesses a distinct tissue-specific enhancer property by itself (Fig 3D' and 3I'), indicating that different function can be evolved independently by multiple TEs located close to one another. In general, hundreds or more of TE types (subfamilies) constitute 20–50% of vertebrate genomes [44]. For example, it has been reported that several TEs contain motifs of functional sequences such as the CTCF-binding motif in rodent B2 SINE [45, 46] or in the MER20 DNA transposon [47], the Nfi-binding motifs in MER130 [48], and the OCT4-binding site in LTR7 or MER74A [49, 50], clearly demonstrating that certain TEs have the potential to acquire a function during evolution. Therefore, by multiple TEs being inserted close to one another, it is possible that they subsequently acquired a new regulatory function during evolution. We expect that many such coordinated TE-derived enhancers are hidden in mammalian genomes. To find clues that support this hypothesis, we searched the human genome for AmnSINE1 copies located proximal to other TEs, all of which overlap CNEs (S3 Table). Among the 626 conserved AmnSINE1 loci, 54 elements (8.6%) accompany other TEs that have been evolutionarily conserved, including all the major TE classes such as SINEs, LINEs, LTR-retrotransposons, and DNA transposons. Thus, the possibility arises that, at least at some loci among these 54 CNEs, several TEs located proximal to one another cooperate to modulate *cis*-regulatory networks that have been involved in the evolution of morphological innovations. This perspective extends the potential of TEs as genetic sources of a broader diversity of *cis*-regulatory elements. Further functional analysis of these TE-derived *cis*-regulatory elements will enhance our understanding of their involvement in morphological innovation during evolution.

## Methods

### Ethics

The mouse strains B6C3F1, C57BL/6, and ICR were purchased from Sankyo Laboratory Service Corporation (Tokyo, Japan). Animals were kept in ventilated cages under a 12-h light/dark cycle at 24°C. This study was approved by the Ethics Committee of Tokyo Institute of Technology and Institutional Animal Care and Use Committee of RIKEN Kobe Branch.

### Transgene construction

A 2.1-kb DNA fragment of the mouse AS3_9 locus was amplified by PCR using primers AS3_9-F and AS3_9-R (S2 Table) containing *Hin*d III recognition sites. The product was cloned into the *Hin*d III site of plasmid HSF51 harboring the mouse heat-shock protein 68 promoter followed by the bacterial *lacZ* reporter gene and the SV40 poly-A signal, yielding the AS3_9-HSF51 construct.

The AS3_9 deletion constructs containing various combinations of TEs (Fig 3A–3I) were generated by overlap extension PCR as described [26]. Briefly, internal primers overlapping complementary sequences (S1 Table) were designed to carry out the deletion of each TE region. The first PCR was performed with one of the internal primers and either one of the vector primers (HSF51-F or HSF51-R; S2 Table) using AS3_9-HSF51 as template. The resulting PCR fragments were used as templates for the second PCR with the two vector primers, and the PCR products were cloned into HSF51 upstream of the heat-shock protein 68 promoter via *Sal* I and *Hin*d III sites.

### Transgenic mouse enhancer assay

Transgenic mice were produced as described [23, 26]. Briefly, the constructs were linearized with *Sal* I and *Xho* I. After purification using the Gel Extraction kit from Qiagen, the DNA fragments were dialyzed against microinjection buffer (5 mM Tris-HCl, 0.1 mM EDTA) at 4°C overnight. Pronuclear microinjection was performed using 6–10 ng/μl of the DNA solution into a B6C3F1 zygote, and microinjected zygotes were transferred to the oviduct of pseudopregnant ICR females.

Transgenic mouse embryos were identified by PCR genotyping using primers LZgt-F02 and LZgt-R01 from yolk samples. The transgenic embryos were fixed for 1 h in phosphate-buffered saline (PBS) containing 1% formaldehyde, 0.1% glutaraldehyde, and 0.05% (v/v) NP-40 and then stained with PBS containing 500 μg/ml X-gal, 5 mM K_3_Fe(CN)_6_, 5 mM K_4_Fe(CN)_6_, 2 mM MgCl_2_, 0.02% NP-40, and 0.01% sodium deoxycholate for >3 h at 37°C. Consistency among *lacZ* expression patterns was confirmed by multiple microinjection experiments. For staining sections, the fixed embryos were permeated with 30% sucrose in PBS overnight at 4°C and embedded with O.C.T. compound (Tissue-Tek, Sakura, Torrance, CA) for sectioning. Coronal sections produced with a cryostat (Leica CM 1850) were counterstained with kernechtrot (Nuclear Fast Red). In addition to the analysis of transient transgenic embryos, we generated three stable lines derived from AS3_9-*lacZ* transgenic mice (S3B Fig). The E9.5–15.5 embryos of each heterozygote transgenic mouse were harvested and stained with X-gal solution as described above.

### ISH

Mouse embryos for ISH for *wnt5a* and *lacZ* were prepared from ICR and stable transgenic lines, respectively. E11.5 embryos for whole-mount ISH were fixed overnight in 4% paraformaldehyde dissolved in diethylpyrocarbonate-treated PBS at 4°C. For section ISH (E13.5–15.5), embryos were permeated overnight with 30% sucrose dissolved in diethylpyrocarbonate-treated PBS after fixation. Then, the embryos were embedded with O.C.T. compound and frozen. Sections were prepared on a Leica sledge microtome at 14 μm and individually mounted on slides. Digoxigenin-labelled antisense RNA probes were synthesized from linearized plasmids with T3 and T7 polymerase (Roche, Basel, Switzerland). Respective plasmids carrying subcloned coding regions of *wnt5a* and *lacZ* were prepared. ISH was performed as described [51].

ISH was also performed on E13.5 coronal cryosections as described [25] to examine the expression of the AS3_9-proximal genes. Briefly, eight genes (*wnt5a*, *erc2*, *lrtm1*, *caca2d3*, *selk*, *actr8*, *il17rb*, and *chdh*) surrounding AS3_9 were identified with the UCSC Genome Browser (http://genome.ucsc.edu/). The cDNAs were amplified by PCR from an E14 or E17 cDNA pool, cloned into pGEM T-easy vectors (Promega, Madison, WI, USA), and used for probe syntheses. Probes prepared by *in vitro* transcription using the DIG RNA Labeling kit (Roche) were purified by lithium chloride precipitation and used for ISH as described [25].

For the ISH of the AS3_9-ko mice for the *wnt5a* probe, the plasmid containing the mouse *wnt5a* cDNA (1.4 kb) was generated via PCR with the primers in S2 Table and sub-cloned into pBluescript (KS-). The embryos were fixed, embedded in Paraplast, and serially sectioned (10 μm thickness). Sections were subjected to ISH as described [51, 52].

### AS3_9-deficient mice

The AS3_9 sequence (chr14:29028538–29029337 of GRCm38/mm10), including the three TEs, was targeted (S6A Fig). Two arm fragments (7.1 kb of the long arm and 3.6 kb of the short arm) were amplified using an LA PCR kit (Takara, Japan) with the following primers: AS3_9-LongArm-F2 and AS3_9-LongArm-R2 for the long arm, and AS3_9-ShortArm-F and AS3_9-ShortArm-R for the short arm (S2 Table). The long-arm fragment was sequenced and cloned into the 5’ (*Not* I and *Sal* I) cloning site of the PGK-Neo-pA cassette in the Targeting vector (DT-A-pA/loxP/PGK-Neo-pA/loxP; see http://www2.clst.riken.jp/arg/cassette.html for details); the short arm was also sequenced and cloned in the 3’ (*Xho* I) site in the same vector. Homologous recombination was conducted using TT2 embryonic stem cells [53], and two recombinants were used to produce chimeric AS3_9-deficient mice (#49 and #154)

For Southern hybridization, DNA probes for the 5’ and 3’ regions of the targeted sequence as well as the Neo sequence were amplified with specific primers (S2 Table) and labelled with [α-^32^P]dCTP. Genomic DNA (10 μg each) from F1 individuals was digested with *Bln* I and *Bsp*1407 I (for the 5’ probe), *Eco*R V (for the 3’ probe), or *Sac* I (for the Neo probe). Signals of 12.0, 6.2, and 6.3 kb for the mutant alleles were detected by Southern blotting using the 5’, 3’, and Neo probes, respectively (S6C–S6E Fig). PCR genotyping was performed with AS3_9-gtF1(KO) and AS3_9-gtR for detection of the knockout allele and AS3_9-gtF1(WT) and AS3_9-gtR for the wild-type allele (S6B Fig; S2 Table).

### Yeast one-hybrid screen

The Matchmaker Gold Yeast One-Hybrid Library Screening System kit (Clontech, Palo Alto, CA, USA) was used to identify proteins that could bind to the AS3_9 sequence. A SMART cDNA library was constructed using the kit from the frontonasal tissues of 21 mouse embryos at E14.5. The bait DNA (chr14:29028539–29029109 of GRCm38/mm10) consisted of AmnSINE1 and X6b_DNA regions because the sequence is responsible for the enhancer activity in maxillary processes, the origin of PS outgrowth. Yeast one-hybrid screening was conducted with two consecutive selection steps with 100 ng/ml (first step) and 500 ng/ml (second step) of the antibiotic aureobasidin A. Among 11.4 million clones of the library screened, 14 positive clones (S1 Table) were isolated consisting of 12 genes in total, which included craniofacial developmental genes (Msx1, Msx2, and Gtf2ird1). Mutation in the Msx1-binding site of the bait sequence was introduced (TAATTGG -> gccgTGt) using appropriate primers (S2 Table), and the yeast one-hybrid assay was performed according to manufacturer's protocol with aureobasidin A (250 ng/ml).

### Candidates for other cooperatively functional CNEs derived from multiple TEs including AmnSINE1

We performed an *in silico* screen of the human genome (hg38) to identify AmnSINE1-derived CNEs proximal to other TE-derived CNEs. By comparing a list of conserved elements (phastConsElements100way) from the UCSC genome database and the latest TE annotation list by RepeatMasker (with the repeat library 20140131; http://www.repeatmasker.org/species/hg.html), all TEs overlapping >30 bp with the conserved elements (LOD score >100) were extracted. Among the 626 evolutionarily conserved AmnSINE1 sequences found, those proximal to (<600 bp) another TE-derived CNE were collected and listed in S3 Table.

### Accession numbers

Details of the AS3_9-ko lines are available at http://www2.clst.riken.jp/arg/mutant%20mice%20list.html (Accession No. CDB0941K).

## Supporting Information

S1 Fig Closure of the secondary palate during mouse development. Upper jaws (A) and coronal sections (B) are illustrated for E13.5–15.5 embryos. Palatal shelves are shown in magenta.
(TIF)

S2 Fig Sequence alignment of the AmnSINE1 region of AS3_9 corresponding to nucleotide positions 391–501 of its consensus sequence. The top line of the alignment shows the consensus sequences of AmnSINE1.
(TIF)

S3 Fig X-gal staining of AS3_9-*lacZ* transgenic embryos. Embryos from the transient enhancer analysis (A) and the three stable lines α–γ (B) at E9.5–15.5 were stained. Scale bar: 2 mm.
(TIF)

S4 Fig ISH of E13.5 coronal sections for the eight genes surrounding AS3_9. (A) *wnt5a*, (B) *erc2*, (C) *lrtm1*, (D) *cacna2d3*, (E) *selk*, (F) *actr8*, (G) *il17rb*, and (H) *chdh*. Red arrowheads denote *wnt5a* expression in palatal shelves.
(TIF)

S5 Fig Comparison of *lacZ* and *wnt5a* expression at E10.5, E13.5, and E14.5. X-gal–stained AS3_9-*lacZ* embryos (A,D), and ISH for *lacZ* (B,E) and *wnt5a* (C,F). Arrowheads: frontonasal region. Ventral view of the X-gal–stained upper jaw of AS3_9-*lacZ* (G,H), coronal sections of the X-gal–stained AS3_9-*lacZ* embryos (I,J), and ISH for *wnt5a* in coronal sections (K,L). Arrowheads: palatal shelves.
(TIF)

S6 Fig Generation of AS3_9-deficient mice. (A) Schematic representation of wild-type and mutant alleles as well as the targeting vector. The 800-bp region containing the three TEs in AS3_9 was replaced with a Neo cassette (Pr-Neo-pA). Black bars: 5’ and 3’ probe regions used for Southern hybridization. Bl: *Bln* I, Bs: *Bsp*1407 I, E: *Eco*R V, and S: *Sac* I sites. (B) Positions of the genotyping primers and PCR product lengths for wild-type and mutant alleles. (C–E) Southern hybridization of the F1 heterozygote AS3_9-ko mice with 5’ (C), 3’ (D), and Neo sequence (E) probes. Five and three individuals of the two independent lines (#49: lanes 1–4 and 8, #154: lanes 5–7) were used, respectively, as well as a wild-type mouse (control; lane 9).
(TIF)

S7 Fig **Coronal sections (10 μm) of E14.5 (A) and E15.5 (B) embryos of AS3_9-ko mice were stained with hematoxylin and eosin.** The embryos were compared among two to five littermates from eight different litters. WT: Wild type. Scale bar: 0.5 mm.
(TIF)

S8 Fig **Sequence alignments of the X6b_DNA (A), and MER117 (B) regions of AS3_9.** The top line of each alignment is the consensus sequences of the TE. The putative Msx1-binding site (reverse-complement of the TAATTG motif) is denoted by a red line in (A).
(TIF)

S9 Fig Representative X-gal–stained E13.5 transgenic embryos. (a–i) Embryos harboring the AS3_9 construct (a) and related deletion constructs (b–i), corresponding to those in Fig 3A–3I. (j) Transgenic embryos harboring the AS3_9 construct in which a mutation was introduced in the putative Msx1-binding site, corresponding to those in Fig 3J.
(TIF)

S10 Fig AS3_9-binding proteins and enhancer activity of the construct containing a mutation in the Msx1-binding site. (A–C) Binding of Msx1, Msx2, Gtf2ird1, and Zbtb7c to the AS3_9 sequence detected by yeast one-hybrid assay using SD/-Leu medium with (B) or without (C) antibiotic (aureobasidin A). Empty vector was used as the negative control. (D–F) Yeast one-hybrid assay was used to assess the viability of Msx1-binding-site mutants using SD/-Leu medium with (E) or without (F) aureobasidin A.
(TIF)

S11 Fig **Epigenetic marks in AS3_9 from the ENCODE (A) and the Roadmap Epigenomics (B) projects.** (A) The human AS3_9 region in the UCSC genome browser (hg19) showing the DNase-hypersensitive clusters and the transcription factor binding signals by the ChIP-seq analyses from the ENCODE project. (B) ChIP-seq or DNase-hypersensitive signals with scores >10 were heat-mapped for each tissues/cells. Maximum scores in each TE region were retrieved form the Roadmap Epigenomics genome browser.
(TIF)

S1 Table Isolated positive clones by yeast one-hybrid screening. In total 14 positive clones contained 12 genes, some of which are included in the same clones.
(DOCX)

S2 Table Primer list used in this study. (XLSX)

S3 Table List of AmnSINE1-derived CNEs accompanied by another repetitive elements under purifying selection. The Locus ID 12 corresponds to AS3_9 enhancer.
(XLSX)
