# Supplementary figures and images for: Coordinately Co-opted Multiple Transposable Elements Constitute an Enhancer for wnt5a Expression in the Mammalian Secondary Palate
Source: PLoS Genet. 2016 Oct 14;12(10):e1006380. doi: 10.1371/journal.pgen.1006380 (PMC5065162; doi:10.1371/journal.pgen.1006380)

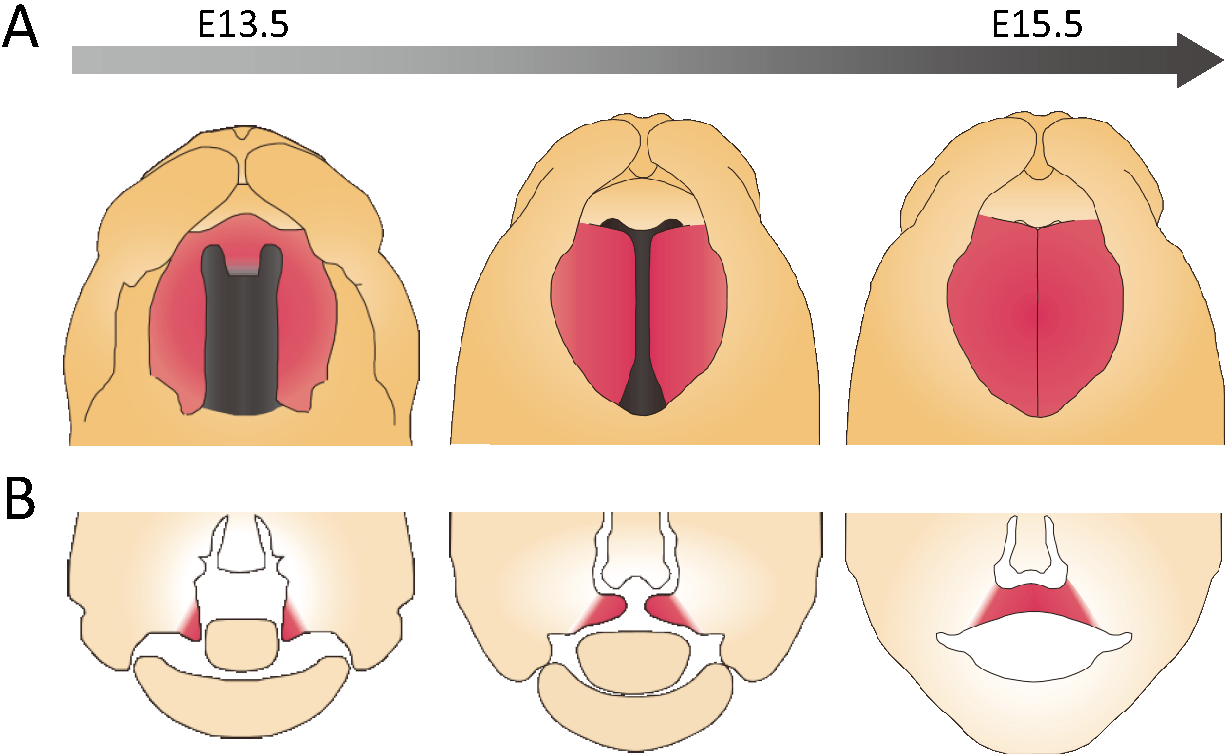

Supplement: S1 Fig — Upper jaws (A) and coronal sections (B) are illustrated for E13.5–15.5 embryos. Palatal shelves are shown in magenta. (TIF) [file pgen.1006380.s001.tif]

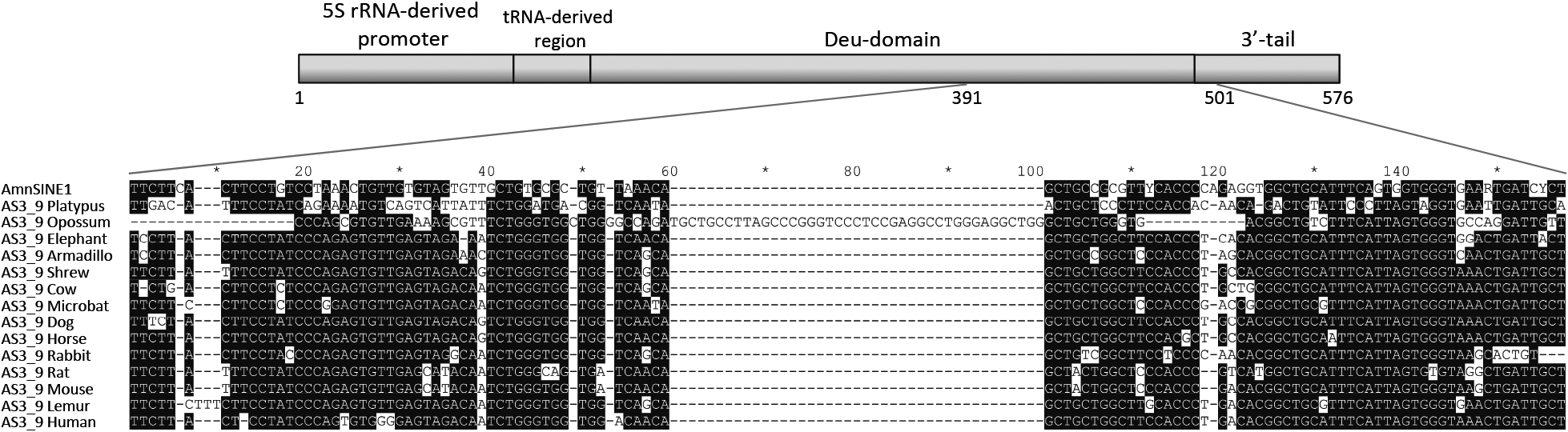

Supplement: S2 Fig — The top line of the alignment shows the consensus sequences of AmnSINE1. (TIF) [file pgen.1006380.s002.tif]

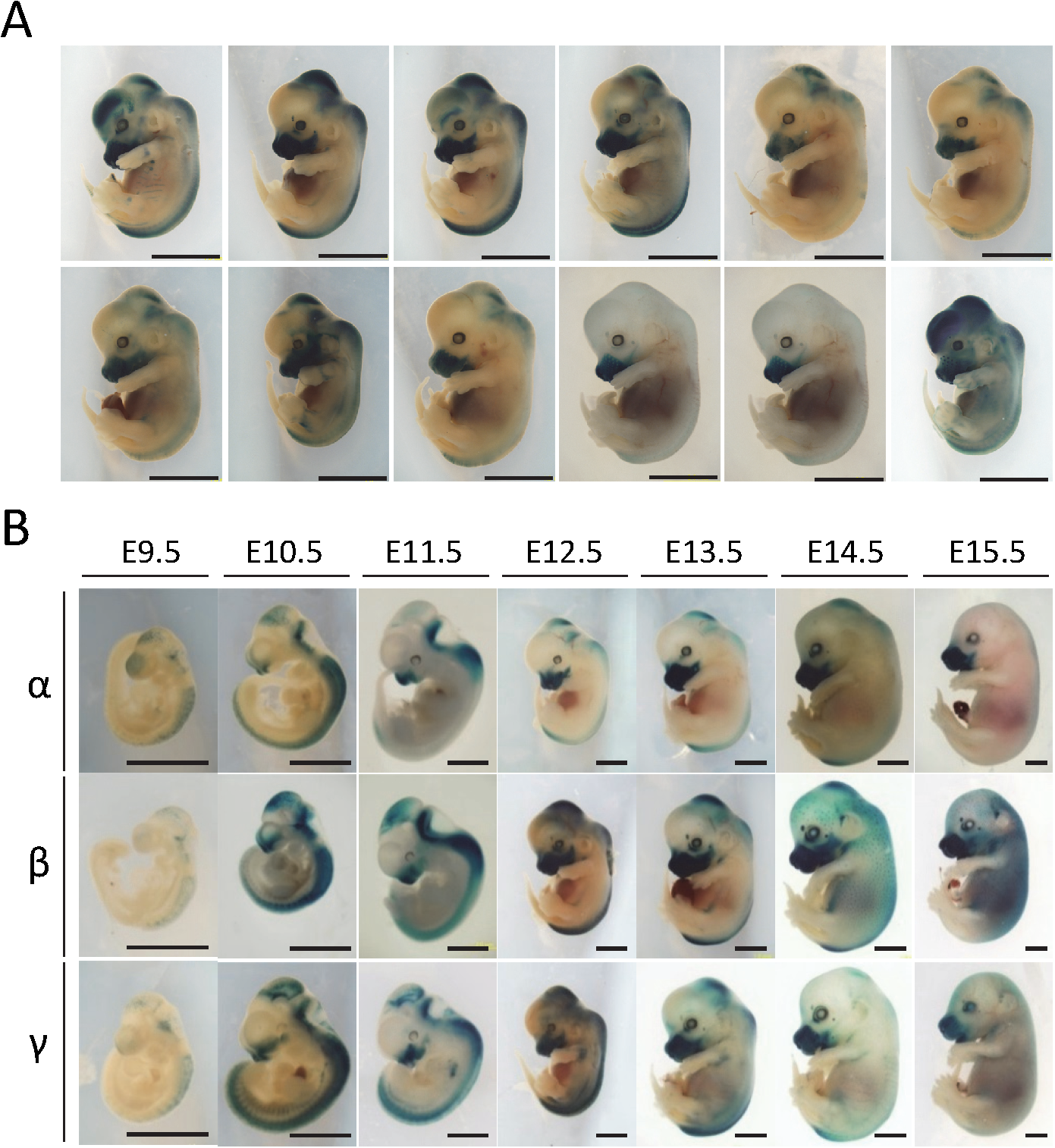

Supplement: S3 Fig — Embryos from the transient enhancer analysis (A) and the three stable lines α–γ (B) at E9.5–15.5 were stained. Scale bar: 2 mm. (TIF) [file pgen.1006380.s003.tif]

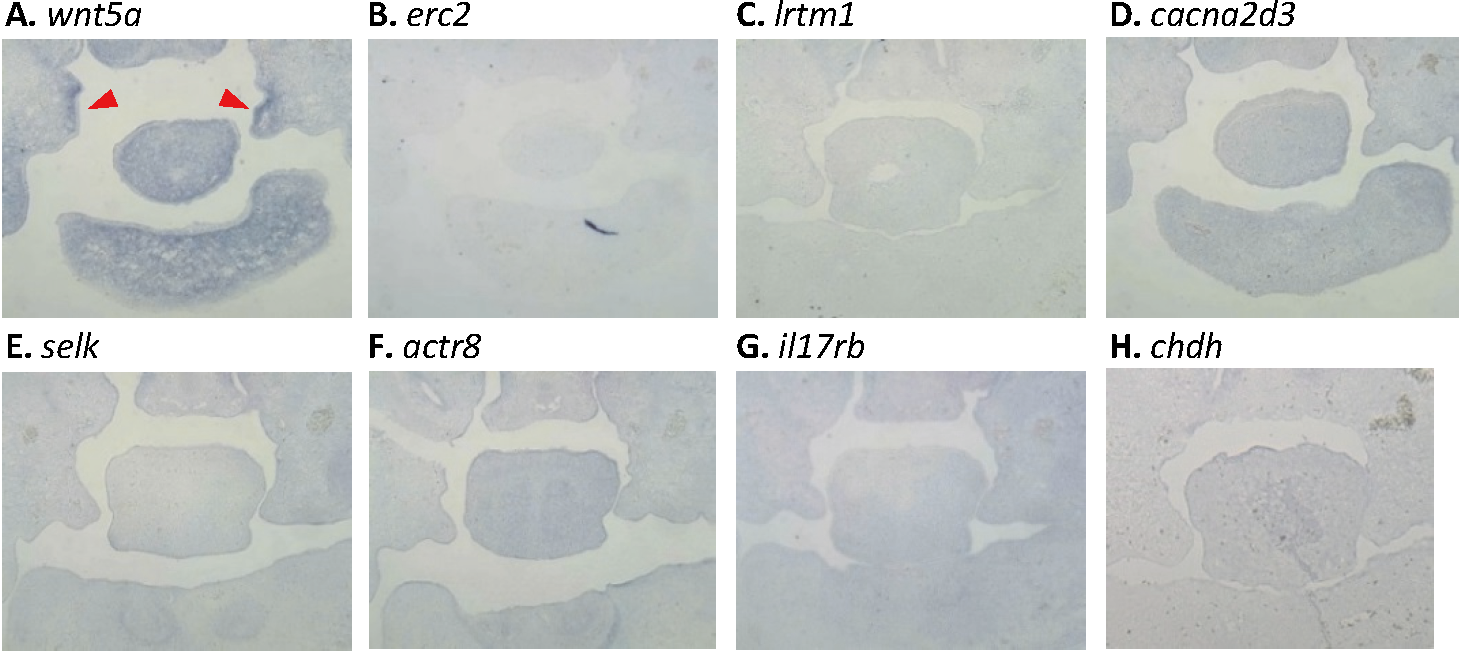

Supplement: S4 Fig — (A) wnt5a, (B) erc2, (C) lrtm1, (D) cacna2d3, (E) selk, (F) actr8, (G) il17rb, and (H) chdh. Red arrowheads denote wnt5a expression in palatal shelves. (TIF) [file pgen.1006380.s004.tif]

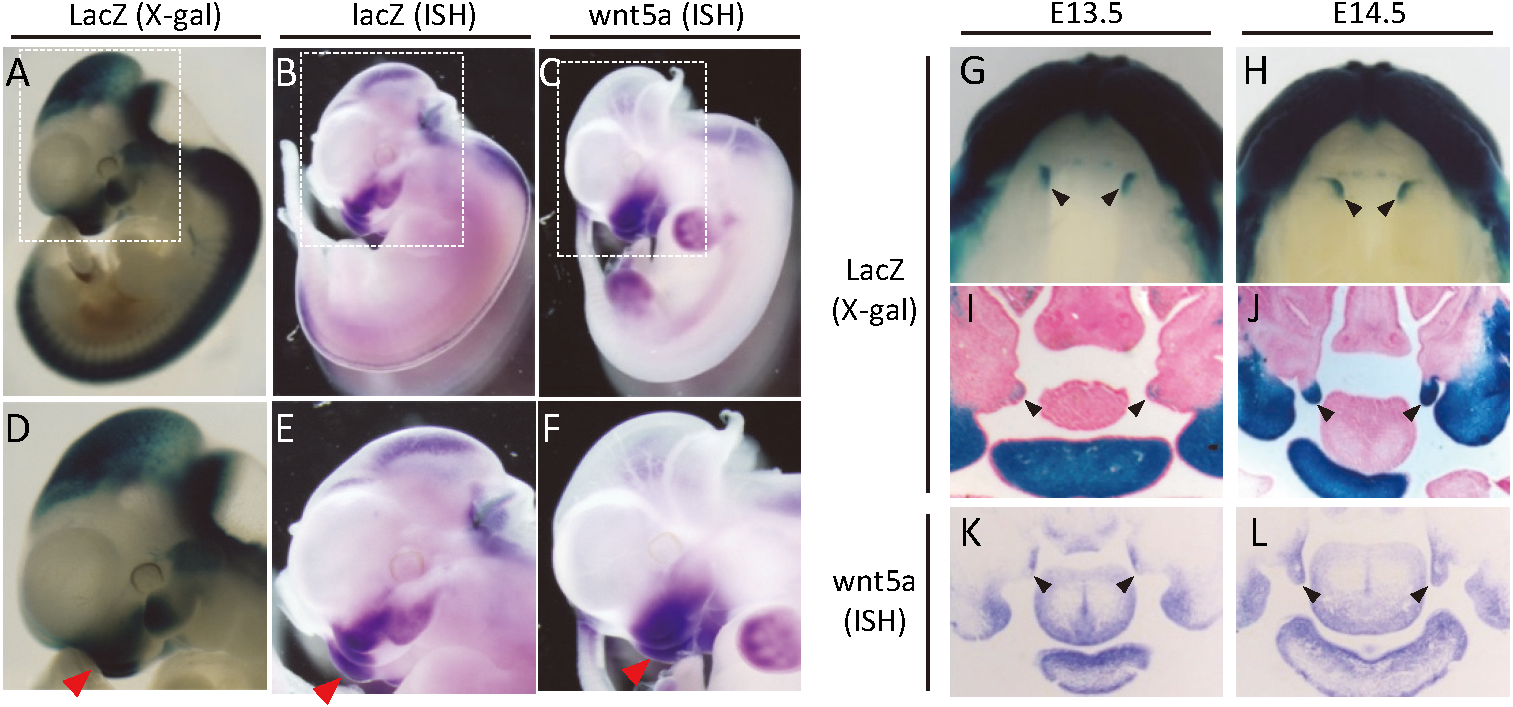

Supplement: S5 Fig — X-gal–stained AS3_9-lacZ embryos (A,D), and ISH for lacZ (B,E) and wnt5a (C,F). Arrowheads: frontonasal region. Ventral view of the X-gal–stained upper jaw of AS3_9-lacZ (G,H), coronal sections of the X-gal–stained AS3_9-lacZ embryos (I,J), and ISH for wnt5a in coronal sections (K,L). Arrowheads: palatal shelves. (TIF) [file pgen.1006380.s005.tif]

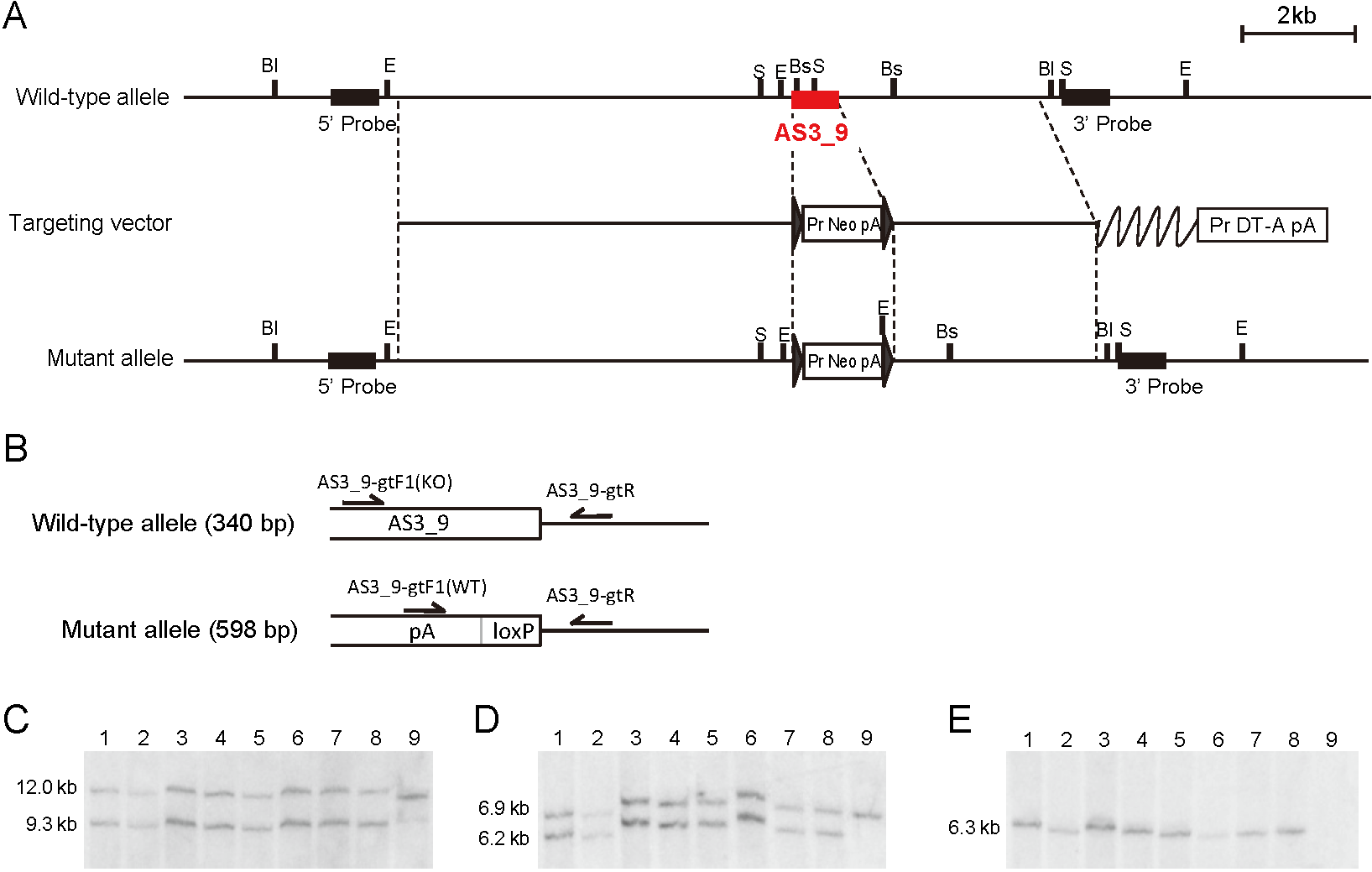

Supplement: S6 Fig — (A) Schematic representation of wild-type and mutant alleles as well as the targeting vector. The 800-bp region containing the three TEs in AS3_9 was replaced with a Neo cassette (Pr-Neo-pA). Black bars: 5’ and 3’ probe regions used for Southern hybridization. Bl: Bln I, Bs: Bsp1407 I, E: EcoR V, and S: Sac I sites. (B) Positions of the genotyping primers and PCR product lengths for wild-type and mutant alleles. (C–E) Southern hybridization of the F1 heterozygote AS3_9-ko mice with 5’ (C), 3’ (D), and Neo sequence (E) probes. Five and three individuals of the two independent lines (#49: lanes 1–4 and 8, #154: lanes 5–7) were used, respectively, as well as a wild-type mouse (control; lane 9). (TIF) [file pgen.1006380.s006.tif]

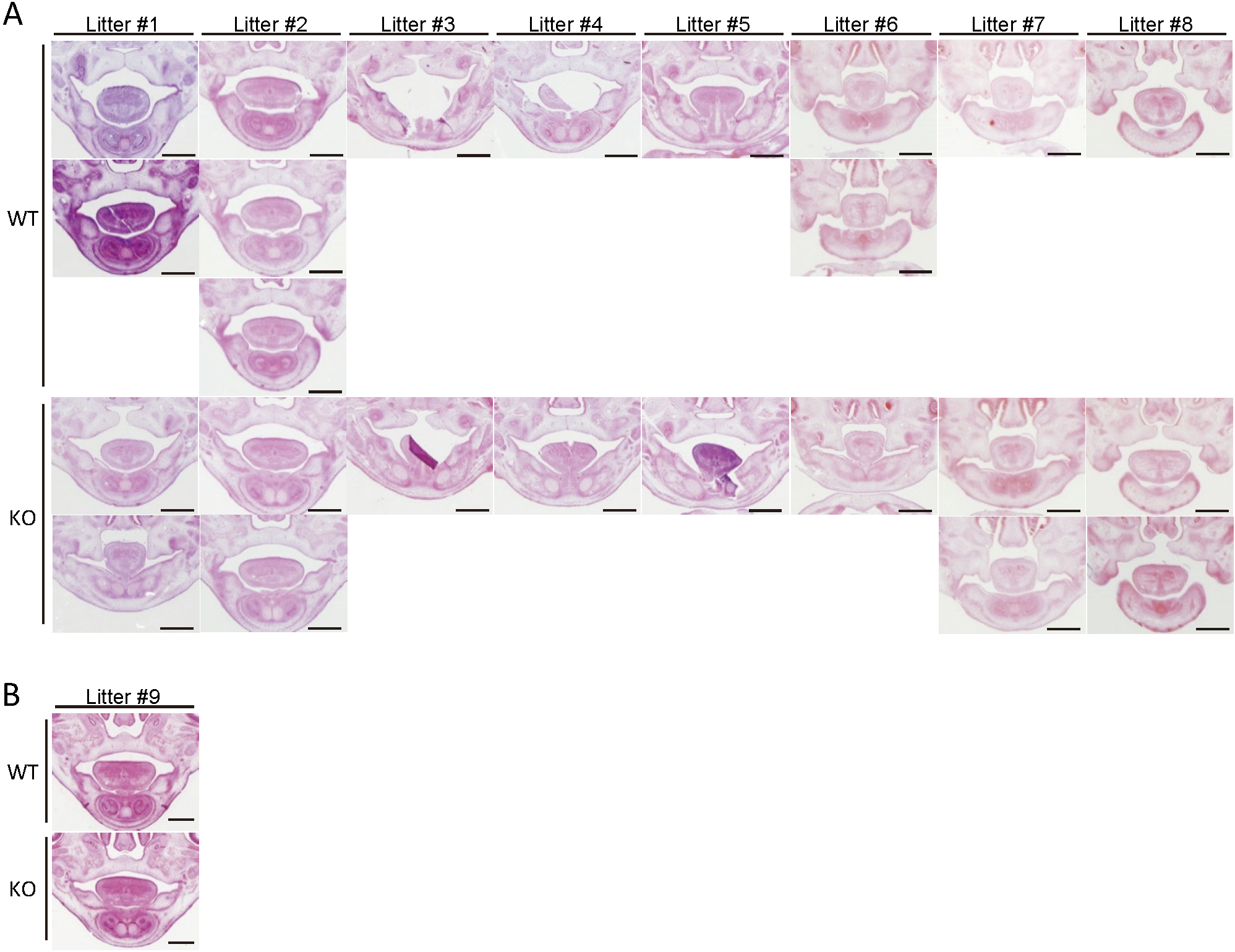

Supplement: S7 Fig — Coronal sections (10 μm) of E14.5 (A) and E15.5 (B) embryos of AS3_9-ko mice were stained with hematoxylin and eosin. The embryos were compared among two to five littermates from eight different litters. WT: Wild type. Scale bar: 0.5 mm. (TIF) [file pgen.1006380.s007.tif]

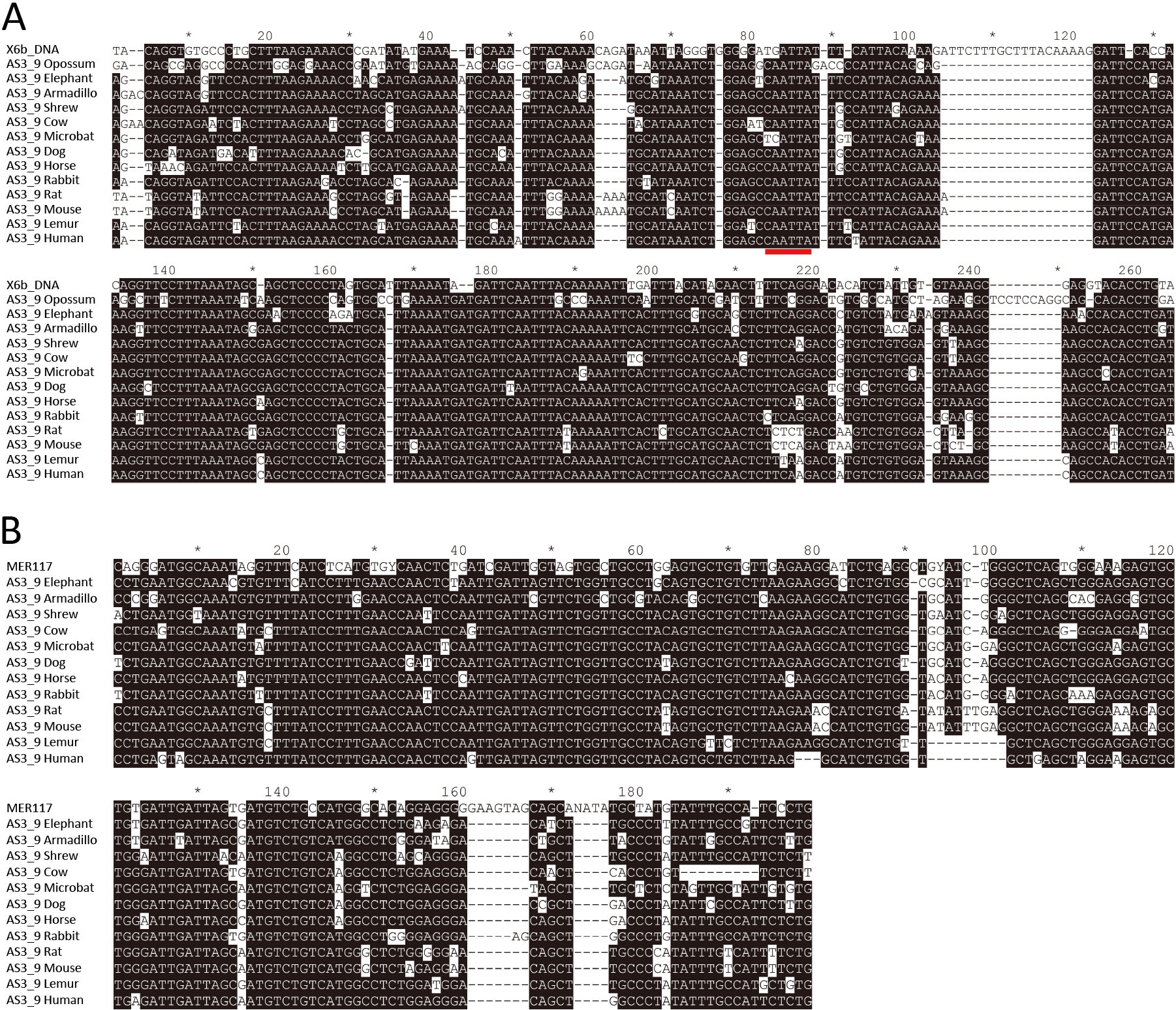

Supplement: S8 Fig — Sequence alignments of the X6b_DNA (A), and MER117 (B) regions of AS3_9. The top line of each alignment is the consensus sequences of the TE. The putative Msx1-binding site (reverse-complement of the TAATTG motif) is denoted by a red line in (A). (TIF) [file pgen.1006380.s008.tif]

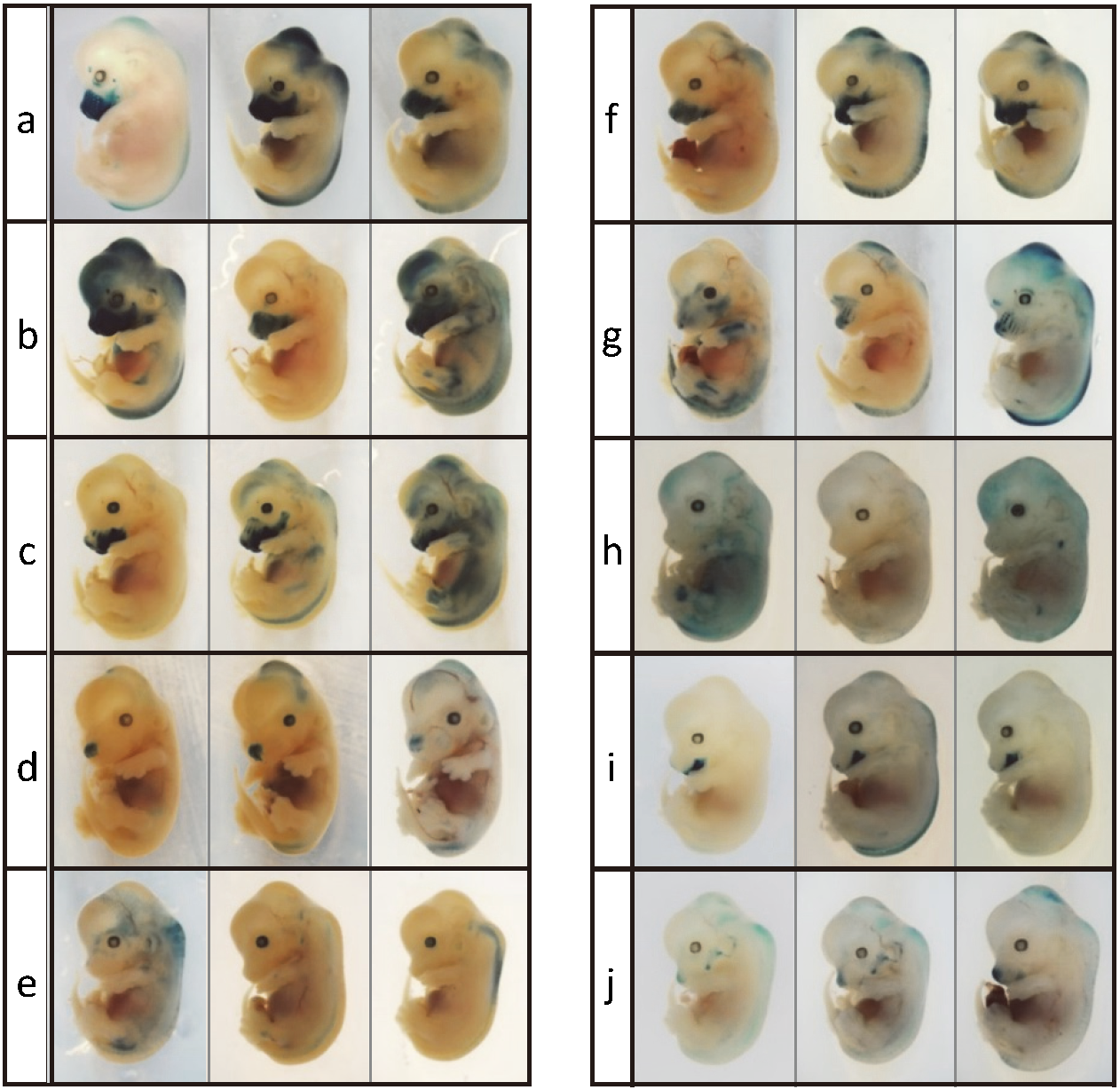

Supplement: S9 Fig — (a–i) Embryos harboring the AS3_9 construct (a) and related deletion constructs (b–i), corresponding to those in Fig 3A–3I. (j) Transgenic embryos harboring the AS3_9 construct in which a mutation was introduced in the putative Msx1-binding site, corresponding to those in Fig 3J. (TIF) [file pgen.1006380.s009.tif]

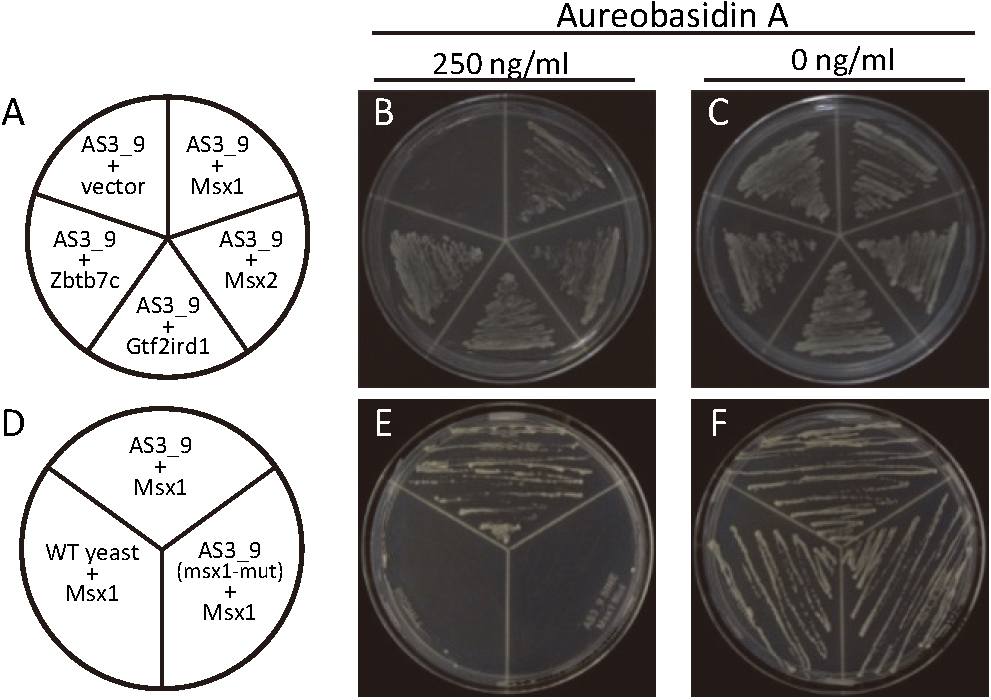

Supplement: S10 Fig — (A–C) Binding of Msx1, Msx2, Gtf2ird1, and Zbtb7c to the AS3_9 sequence detected by yeast one-hybrid assay using SD/-Leu medium with (B) or without (C) antibiotic (aureobasidin A). Empty vector was used as the negative control. (D–F) Yeast one-hybrid assay was used to assess the viability of Msx1-binding-site mutants using SD/-Leu medium with (E) or without (F) aureobasidin A. (TIF) [file pgen.1006380.s010.tif]

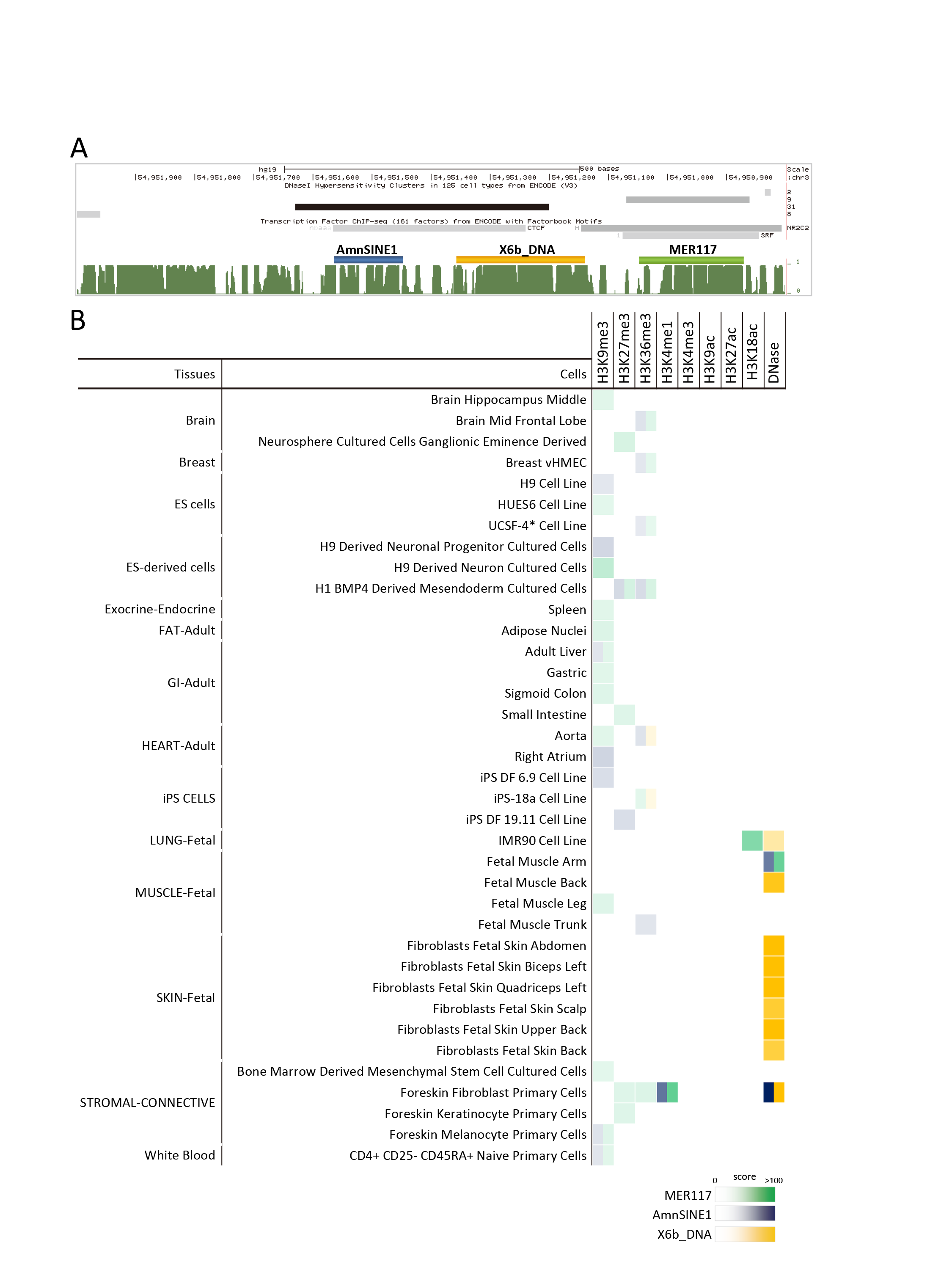

Supplement: S11 Fig — Epigenetic marks in AS3_9 from the ENCODE (A) and the Roadmap Epigenomics (B) projects. (A) The human AS3_9 region in the UCSC genome browser (hg19) showing the DNase-hypersensitive clusters and the transcription factor binding signals by the ChIP-seq analyses from the ENCODE project. (B) ChIP-seq or DNase-hypersensitive signals with scores >10 were heat-mapped for each tissues/cells. Maximum scores in each TE region were retrieved form the Roadmap Epigenomics genome browser. (TIF) [file pgen.1006380.s011.tif]
