# Supplementary material for: Coordinately Co-opted Multiple Transposable Elements Constitute an Enhancer for wnt5a Expression in the Mammalian Secondary Palate
Source: PLoS Genet. 2016 Oct 14;12(10):e1006380. doi: 10.1371/journal.pgen.1006380 (PMC5065162; doi:10.1371/journal.pgen.1006380)
Supplement: S1 Table — In total 14 positive clones contained 12 genes, some of which are included in the same clones. (DOCX) [file pgen.1006380.s012.docx]

**S1 Table.** Isolated positive clones by yeast one-hybrid screening.

| **Clone ID** | **Gene** |
| --- | --- |
| **Y39-15** | Ap2m1 (Adaptor-related protein complex 2, mu 1 subunit) |
| **Y39-37** | Dhx34 (DEAH (Asp-Glu-Ala-His) box polypeptide 34) |
| **Y39-39** | Maz (MYC-associated zinc finger protein) |
| **Y39-45** | Zbtb7c (Zinc finger and BTB domain containing 7C) |
| **Y39-48** | RBBP7 (Retinoblastoma binding protein 7)  Gtf2ird1 (General transcription factor II I repeat domain-containing 1) |
| **Y39-53** | Msx2 (Msh homeobox 2) |
| **Y39-56** | Dpf3 (Zinc and double PHD fingers, family 3)  Tubb2c (Tubulin beta-4B chain) |
| **Y39-57** | Gtf2ird1 |
| **Y39-61** | Vezf1 (Vascular endothelial zinc finger 1)  Polr2c (Polymerase (RNA) II (DNA directed) polypeptide C) |
| **Y39-84** | Msx1 (Msh homeobox 1) |
| **Y39-92** | Zbtb7c |
| **Y39-108** | Zbtb7c |
| **Y39-113** | Gtf2ird1 |
| **Y39-166** | Zbtb7c |

In total 14 positive clones contained 12 genes, some of which are included in the same clones.
